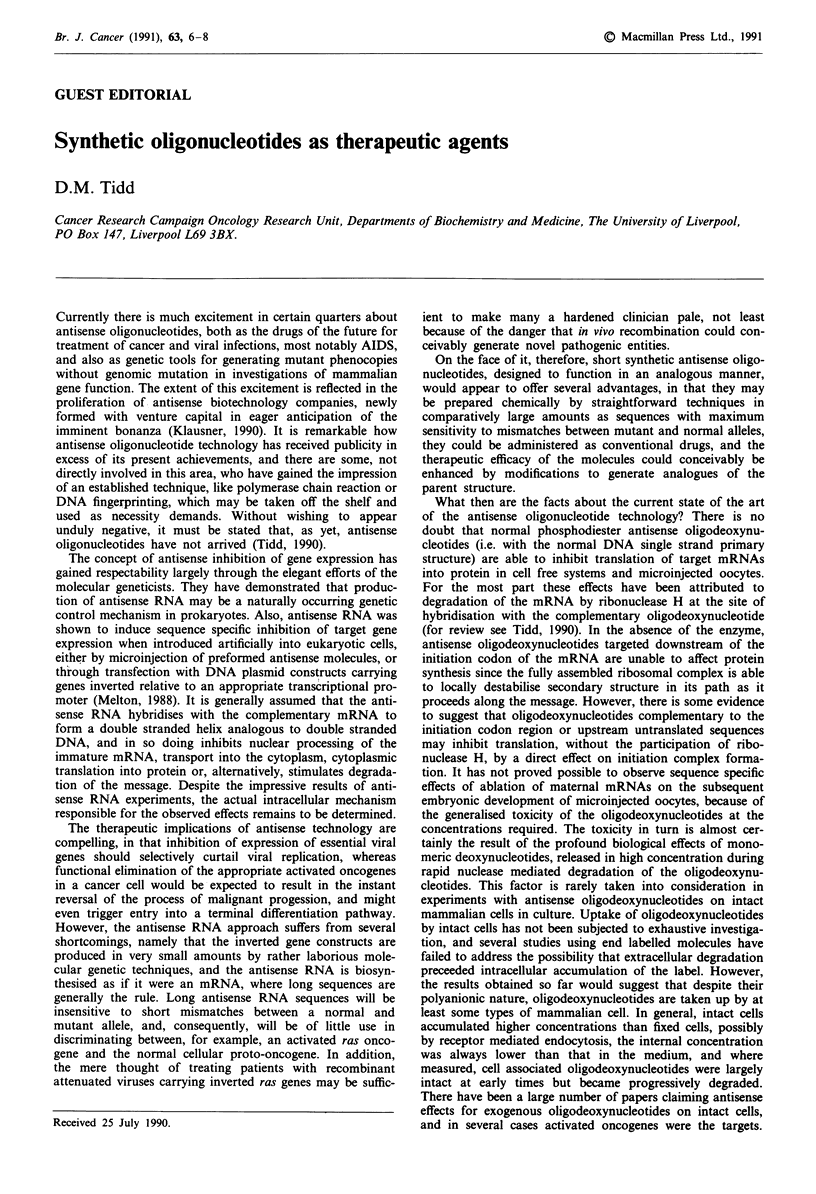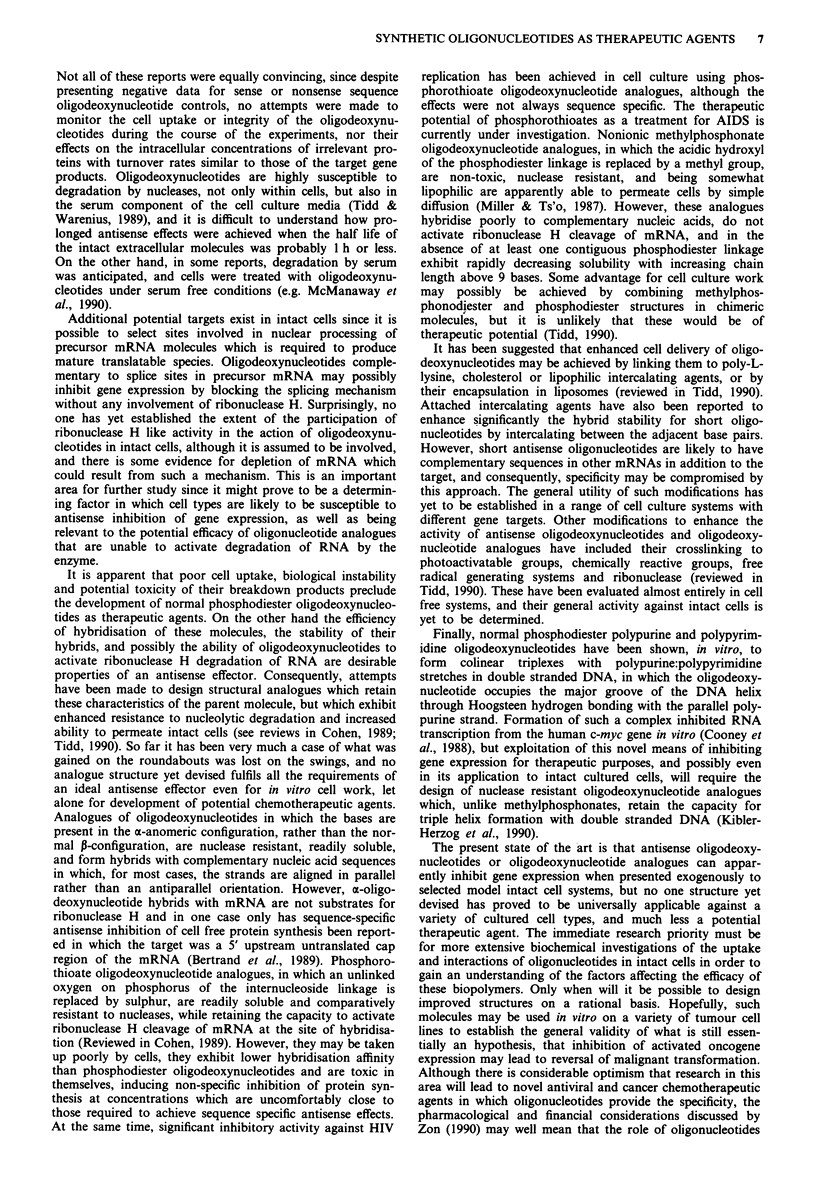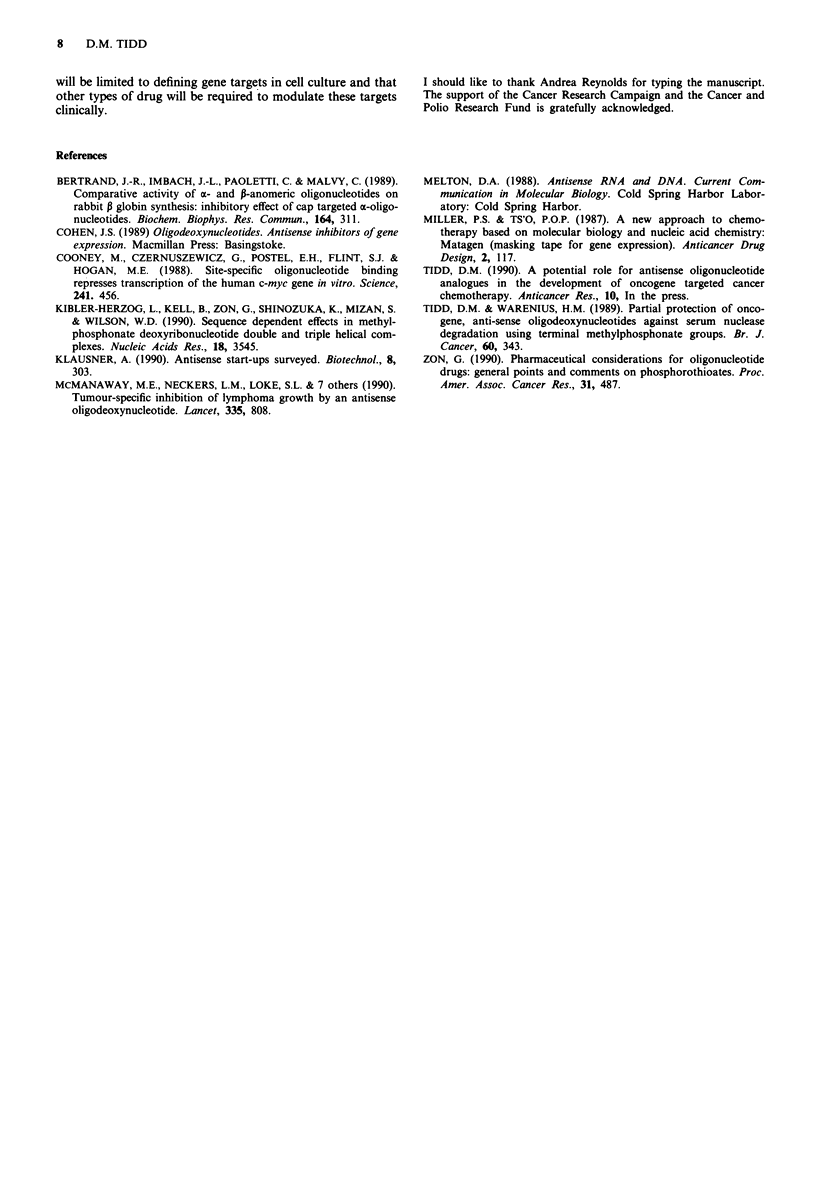# Synthetic oligonucleotides as therapeutic agents.

**DOI:** 10.1038/bjc.1991.3

**Published:** 1991-01

**Authors:** D. M. Tidd


Br. J. Cancer (1991), 63, 6-8 © Macmillan Press Ltd., 1991

GUEST EDITORIAL

### Synthetic oligonucleotides as therapeutic agents

D.M. Tidd

*Cancer Research Campaign Oncology Research Unit, Departments of Biochemistry and Medicine, The University of Liverpool, PO Box 147, Liverpool L69 3BX.*

Currently there is much excitement in certain quarters about antisense oligonucleotides, both as the drugs of the future for treatment of cancer and viral infections, most notably AIDS, and also as genetic tools for generating mutant phenocopies without genomic mutation in investigations of mammalian gene function. The extent of this excitement is reflected in the proliferation of antisense biotechnology companies, newly formed with venture capital in eager anticipation of the imminent bonanza (Klausner, 1990). It is remarkable how antisense oligonucleotide technology has received publicity in excess of its present achievements, and there are some, not directly involved in this area, who have gained the impression of an established technique, like polymerase chain reaction or DNA fingerprinting, which may be taken off the shelf and used as necessity demands. Without wishing to appear unduly negative, it must be stated that, as yet, antisense oligonucleotides have not arrived (Tidd, 1990).

The concept of antisense inhibition of gene expression has gained respectability largely through the elegant efforts of the molecular geneticists. They have demonstrated that production of antisense RNA may be a naturally occurring genetic control mechanism in prokaryotes. Also, antisense RNA was shown to induce sequence specific inhibition of target gene expression when introduced artificially into eukaryotic cells, either by microinjection of preformed antisense molecules, or through transfection with DNA plasmid constructs carrying genes inverted relative to an appropriate transcriptional promoter (Melton, 1988). It is generally assumed that the antisense RNA hybridises with the complementary mRNA to form a double stranded helix analogous to double stranded DNA, and in so doing inhibits nuclear processing of the immature mRNA, transport into the cytoplasm, cytoplasmic translation into protein or, alternatively, stimulates degradation of the message. Despite the impressive results of antisense RNA experiments, the actual intracellular mechanism responsible for the observed effects remains to be determined.

The therapeutic implications of antisense technology are compelling, in that inhibition of expression of essential viral genes should selectively curtail viral replication, whereas functional elimination of the appropriate activated oncogenes in a cancer cell would be expected to result in the instant reversal of the process of malignant progession, and might even trigger entry into a terminal differentiation pathway. However, the antisense RNA approach suffers from several shortcomings, namely that the inverted gene constructs are produced in very small amounts by rather laborious molecular genetic techniques, and the antisense RNA is biosynthesised as if it were an mRNA, where long sequences are generally the rule. Long antisense RNA sequences will be insensitive to short mismatches between a normal and mutant allele, and, consequently, will be of little use in discriminating between, for example, an activated *ras* oncogene and the normal cellular proto-oncogene. In addition, the mere thought of treating patients with recombinant attenuated viruses carrying inverted *ras* genes may be sufficient to make many a hardened clinician pale, not least because of the danger that *in vivo* recombination could conceivably generate novel pathogenic entities.

On the face of it, therefore, short synthetic antisense oligonucleotides, designed to function in an analogous manner, would appear to offer several advantages, in that they may be prepared chemically by straightforward techniques in comparatively large amounts as sequences with maximum sensitivity to mismatches between mutant and normal alleles, they could be administered as conventional drugs, and the therapeutic efficacy of the molecules could conceivably be enhanced by modifications to generate analogues of the parent structure.

What then are the facts about the current state of the art of the antisense oligonucleotide technology? There is no doubt that normal phosphodiester antisense oligodeoxynucleotides (i.e. with the normal DNA single strand primary structure) are able to inhibit translation of target mRNAs into protein in cell free systems and microinjected oocytes. For the most part these effects have been attributed to degradation of the mRNA by ribonuclease H at the site of hybridisation with the complementary oligodeoxynucleotide (for review see Tidd, 1990). In the absence of the enzyme, antisense oligodeoxynucleotides targeted downstream of the initiation codon of the mRNA are unable to affect protein synthesis since the fully assembled ribosomal complex is able to locally destabilise secondary structure in its path as it proceeds along the message. However, there is some evidence to suggest that oligodeoxynucleotides complementary to the initiation codon region or upstream untranslated sequences may inhibit translation, without the participation of ribonuclease H, by a direct effect on initiation complex formation. It has not proved possible to observe sequence specific effects of ablation of maternal mRNAs on the subsequent embryonic development of microinjected oocytes, because of the generalised toxicity of the oligodeoxynucleotides at the concentrations required. The toxicity in turn is almost certainly the result of the profound biological effects of monomeric deoxynucleotides, released in high concentration during rapid nuclease mediated degradation of the oligodeoxynucleotides. This factor is rarely taken into consideration in experiments with antisense oligodeoxynucleotides on intact mammalian cells in culture. Uptake of oligodeoxynucleotides by intact cells has not been subjected to exhaustive investigation, and several studies using end labelled molecules have failed to address the possibility that extracellular degradation preceeded intracellular accumulation of the label. However, the results obtained so far would suggest that despite their polyanionic nature, oligodeoxynucleotides are taken up by at least some types of mammalian cell. In general, intact cells accumulated higher concentrations than fixed cells, possibly by receptor mediated endocytosis, the internal concentration was always lower than that in the medium, and where measured, cell associated oligodeoxynucleotides were largely intact at early times but became progressively degraded. There have been a large number of papers claiming antisense effects for exogenous oligodeoxynucleotides on intact cells, and in several cases activated oncogenes were the targets.

Received 25 July 1990.

Not all of these reports were equally convincing, since despite presenting negative data for sense or nonsense sequence oligodeoxynucleotide controls, no attempts were made to monitor the cell uptake or integrity of the oligodeoxynucleotides during the course of the experiments, nor their effects on the intracellular concentrations of irrelevant proteins with turnover rates similar to those of the target gene products. Oligodeoxynucleotides are highly susceptible to degradation by nucleases, not only within cells, but also in the serum component of the cell culture media (Tidd & Warenius, 1989), and it is difficult to understand how prolonged antisense effects were achieved when the half life of the intact extracellular molecules was probably 1 h or less. On the other hand, in some reports, degradation by serum was anticipated, and cells were treated with oligodeoxynucleotides under serum free conditions (e.g. McManaway *et al.*, 1990).

Additional potential targets exist in intact cells since it is possible to select sites involved in nuclear processing of precursor mRNA molecules which is required to produce mature translatable species. Oligodeoxynucleotides complementary to splice sites in precursor mRNA may possibly inhibit gene expression by blocking the splicing mechanism without any involvement of ribonuclease H. Surprisingly, no one has yet established the extent of the participation of ribonuclease H like activity in the action of oligodeoxynucleotides in intact cells, although it is assumed to be involved, and there is some evidence for depletion of mRNA which could result from such a mechanism. This is an important area for further study since it might prove to be a determining factor in which cell types are likely to be susceptible to antisense inhibition of gene expression, as well as being relevant to the potential efficacy of oligonucleotide analogues that are unable to activate degradation of RNA by the enzyme.

It is apparent that poor cell uptake, biological instability and potential toxicity of their breakdown products preclude the development of normal phosphodiester oligodeoxynucleotides as therapeutic agents. On the other hand the efficiency of hybridisation of these molecules, the stability of their hybrids, and possibly the ability of oligodeoxynucleotides to activate ribonuclease H degradation of RNA are desirable properties of an antisense effector. Consequently, attempts have been made to design structural analogues which retain these characteristics of the parent molecule, but which exhibit enhanced resistance to nucleolytic degradation and increased ability to permeate intact cells (see reviews in Cohen, 1989; Tidd, 1990). So far it has been very much a case of what was gained on the roundabouts was lost on the swings, and no analogue structure yet devised fulfils all the requirements of an ideal antisense effector even for *in vitro* cell work, let alone for development of potential chemotherapeutic agents. Analogues of oligodeoxynucleotides in which the bases are present in the α-anomeric configuration, rather than the normal β-configuration, are nuclease resistant, readily soluble, and form hybrids with complementary nucleic acid sequences in which, for most cases, the strands are aligned in parallel rather than an antiparallel orientation. However, α-oligodeoxynucleotide hybrids with mRNA are not substrates for ribonuclease H and in one case only has sequence-specific antisense inhibition of cell free protein synthesis been reported in which the target was a 5′ upstream untranslated cap region of the mRNA (Bertrand *et al.*, 1989). Phosphorothioate oligodeoxynucleotide analogues, in which an unlinked oxygen on phosphorus of the internucleoside linkage is replaced by sulphur, are readily soluble and comparatively resistant to nucleases, while retaining the capacity to activate ribonuclease H cleavage of mRNA at the site of hybridisation (Reviewed in Cohen, 1989). However, they may be taken up poorly by cells, they exhibit lower hybridisation affinity than phosphodiester oligodeoxynucleotides and are toxic in themselves, inducing non-specific inhibition of protein synthesis at concentrations which are uncomfortably close to those required to achieve sequence specific antisense effects. At the same time, significant inhibitory activity against HIV replication has been achieved in cell culture using phosphorothioate oligodeoxynucleotide analogues, although the effects were not always sequence specific. The therapeutic potential of phosphorothioates as a treatment for AIDS is currently under investigation. Nonionic methylphosphonate oligodeoxynucleotide analogues, in which the acidic hydroxyl of the phosphodiester linkage is replaced by a methyl group, are non-toxic, nuclease resistant, and being somewhat lipophilic are apparently able to permeate cells by simple diffusion (Miller & Ts'o, 1987). However, these analogues hybridise poorly to complementary nucleic acids, do not activate ribonuclease H cleavage of mRNA, and in the absence of at least one contiguous phosphodiester linkage exhibit rapidly decreasing solubility with increasing chain length above 9 bases. Some advantage for cell culture work may possibly be achieved by combining methylphosphonodiester and phosphodiester structures in chimeric molecules, but it is unlikely that these would be of therapeutic potential (Tidd, 1990).

It has been suggested that enhanced cell delivery of oligodeoxynucleotides may be achieved by linking them to poly-L-lysine, cholesterol or lipophilic intercalating agents, or by their encapsulation in liposomes (reviewed in Tidd, 1990). Attached intercalating agents have also been reported to enhance significantly the hybrid stability for short oligonucleotides by intercalating between the adjacent base pairs. However, short antisense oligonucleotides are likely to have complementary sequences in other mRNAs in addition to the target, and consequently, specificity may be compromised by this approach. The general utility of such modifications has yet to be established in a range of cell culture systems with different gene targets. Other modifications to enhance the activity of antisense oligodeoxynucleotides and oligodeoxynucleotide analogues have included their crosslinking to photoactivatable groups, chemically reactive groups, free radical generating systems and ribonuclease (reviewed in Tidd, 1990). These have been evaluated almost entirely in cell free systems, and their general activity against intact cells is yet to be determined.

Finally, normal phosphodiester polypurine and polypyrimidine oligodeoxynucleotides have been shown, *in vitro*, to form colinear triplexes with polypurine:polypyrimidine stretches in double stranded DNA, in which the oligodeoxynucleotide occupies the major groove of the DNA helix through Hoogsteen hydrogen bonding with the parallel polypurine strand. Formation of such a complex inhibited RNA transcription from the human c-*myc* gene *in vitro* (Cooney *et al.*, 1988), but exploitation of this novel means of inhibiting gene expression for therapeutic purposes, and possibly even in its application to intact cultured cells, will require the design of nuclease resistant oligodeoxynucleotide analogues which, unlike methylphosphonates, retain the capacity for triple helix formation with double stranded DNA (Kibler-Herzog *et al.*, 1990).

The present state of the art is that antisense oligodeoxynucleotides or oligodeoxynucleotide analogues can apparently inhibit gene expression when presented exogenously to selected model intact cell systems, but no one structure yet devised has proved to be universally applicable against a variety of cultured cell types, and much less a potential therapeutic agent. The immediate research priority must be for more extensive biochemical investigations of the uptake and interactions of oligonucleotides in intact cells in order to gain an understanding of the factors affecting the efficacy of these biopolymers. Only when will it be possible to design improved structures on a rational basis. Hopefully, such molecules may be used *in vitro* on a variety of tumour cell lines to establish the general validity of what is still essentially an hypothesis, that inhibition of activated oncogene expression may lead to reversal of malignant transformation. Although there is considerable optimism that research in this area will lead to novel antiviral and cancer chemotherapeutic agents in which oligonucleotides provide the specificity, the pharmacological and financial considerations discussed by Zon (1990) may well mean that the role of oligonucleotides will be limited to defining gene targets in cell culture and that other types of drug will be required to modulate these targets clinically.

I should like to thank Andrea Reynolds for typing the manuscript. The support of the Cancer Research Campaign and the Cancer and Polio Research Fund is gratefully acknowledged.

### References

BERTRAND, J.-R., IMBACH, J.-L., PAOLETTI, C. & MALVY, C. (1989). Comparative activity of α- and β-anomeric oligonucleotides on rabbit β globin synthesis: inhibitory effect of cap targeted α-oligonucleotides. *Biochem. Biophys. Res. Commun.*, **164**, 311.

COHEN, J.S. (1989) *Oligodeoxynucleotides. Antisense inhibitors of gene expression*. Macmillan Press: Basingstoke.

COONEY, M., CZERNUSZEWICZ, G., POSTEL, E.H., FLINT, S.J. & HOGAN, M.E. (1988). Site-specific oligonucleotide binding represses transcription of the human c-*myc* gene *in vitro*. *Science*, **241**. 456.

KIBLER-HERZOG, L., KELL, B., ZON, G., SHINOZUKA, K., MIZAN, S. & WILSON, W.D. (1990). Sequence dependent effects in methylphosphonate deoxyribonucleotide double and triple helical complexes. *Nucleic Acids Res.*, **18**, 3545.

KLAUSNER, A. (1990). Antisense start-ups surveyed. *Biotechnol.*, **8**, 303.

MCMANAWAY, M.E., NECKERS, L.M., LOKE, S.L. & 7 others (1990). Tumour-specific inhibition of lymphoma growth by an antisense oligodeoxynucleotide. *Lancet*, **335**, 808.

MELTON, D.A. (1988). *Antisense RNA and DNA. Current Communication in Molecular Biology*. Cold Spring Harbor Laboratory: Cold Spring Harbor.

MILLER, P.S. & TS'O, P.O.P. (1987). A new approach to chemotherapy based on molecular biology and nucleic acid chemistry: Matagen (masking tape for gene expression). *Anticancer Drug Design*, **2**, 117.

TIDD, D.M. (1990). A potential role for antisense oligonucleotide analogues in the development of oncogene targeted cancer chemotherapy. *Anticancer Res.*, **10**, In the press.

TIDD, D.M. & WARENIUS, H.M. (1989). Partial protection of oncogene, anti-sense oligodeoxynucleotides against serum nuclease degradation using terminal methylphosphonate groups. *Br. J. Cancer*, **60**, 343.

ZON, G. (1990). Pharmaceutical considerations for oligonucleotide drugs: general points and comments on phosphorothioates. *Proc. Amer. Assoc. Cancer Res.*, **31**, 487.